# A Prescription

**Published:** 1848-01

**Authors:** 


*A Prescription.*—The following prescription, whichwas received at an apothecary's shop in a village in this state, may possess some value as a curious specimen of this branch of literature. We give it *verbatim, et literatim, et punctuatim*, premising, that we have no reason to believe that it was written by one who has any claims to be considered a regular physician. We should not be vastly surprised, however, if the author were a practitioner with not an inconsiderable share of patronage:—

A receit for the Egar 1 pint of hollands gin, 30 grains of Kenine powders 3 cents worth of Jollop and 5 drops of the licks of viteral all compounded up together, take one half A Wine glass 3 times A Day.

